# Super-Elderly Case of Acute Lower Limb Ischemia Treated with Indigo Aspiration System in Japan

**DOI:** 10.3400/avd.cr.23-00095

**Published:** 2024-01-25

**Authors:** Shinsuke Kikuchi, Seima Ohira, Tsutomu Doita, Keisuke Kamada, Naoya Kuriyama, Yuya Tamaru, Takamitsu Tatsukawa, Yuri Yoshida, Daiki Uchida, Nobuyoshi Azuma

**Affiliations:** 1 Department of Vascular Surgery, Asahikawa Medical University, Asahikawa, Hokkaido, Japan; 2 Department of Cardiovascular Surgery, Osaka University, Suita, Osaka, Japan

**Keywords:** acute limb ischemia, Indigo Aspiration System, endovascular therapy

## Abstract

The Indigo Aspiration System (Penumbra Ltd., Alameda, CA, USA), a catheter-based device intended for the endovascular removal of clots from peripheral arteries and veins, was launched in Japan to treat acute limb ischemia after the cessation of urokinase sales. The initial application of this system in Japan was on a 96-year-old male patient. He was diagnosed with acute lower limb ischemia, which was caused by an embolism from a left common iliac artery aneurysm. The treatment significantly enhanced the perfusion to his left foot. This case report elaborates on the patient’s treatment experience and discusses the indications for using the device.

## Introduction

Urokinase, also known as urokinase-type plasminogen activator, is used as a thrombolytic medication to dissolve blood clots. Acute limb ischemia (ALI), venous thromboembolism (VTE), and superior mesenteric artery (SMA) occlusion were commonly treated with the thrombolytic effect of urokinase; however, the sale of urokinase was halted due to its low specific activity in the production process. In Japan, urokinase is covered by Japanese health insurance (≤240000 units/day). However, the use of tissue plasminogen activator is restricted to the treatment of acute myocardial and brain tissue ischemia caused by thrombosis (approved number 20300AMZ00230 by PMDA), and is not approved for ALI, VTE, and SMA occlusion. In this context, four Japanese societies – the Japanese Society for Vascular Surgery (JSVS), the Japanese Society of Phlebology (JSP), the Japanese Association of Cardiovascular Intervention and Therapeutics (CVIT), and the Japanese Society of Interventional Radiology (JSIR) have endeavored to provide an alternative to urokinase in order to maintain the quality of treatment for acute thrombotic events. The focus was on the Indigo Aspiration Device (Penumbra Ltd., Alameda, CA, USA), an endovascularly designed tool for removing clots from arteries and veins in the peripheral vasculature. Endovascular vacuum-assisted thrombectomy devices, similar to those used in managing acute ischemic stroke, are now available for peripheral arteries, such as the Indigo System-Catheter Direct Thrombus Aspiration (IS-CDTA).^1)^ The device has already been approved by the Pharmaceuticals and Medical Devices Agency in Japan in May 2023 (Approval number 30500BZI00017000). It is indicated for acute lower limb ischemia (ALLI), SMA occlusion, and deep venous thrombosis (DVT) when surgical thrombectomy proves ineffective. Five preceding faculties, including our institution, were selected for trials prior to postmarketing surveillance.

## Case Report

**Figure 1:**
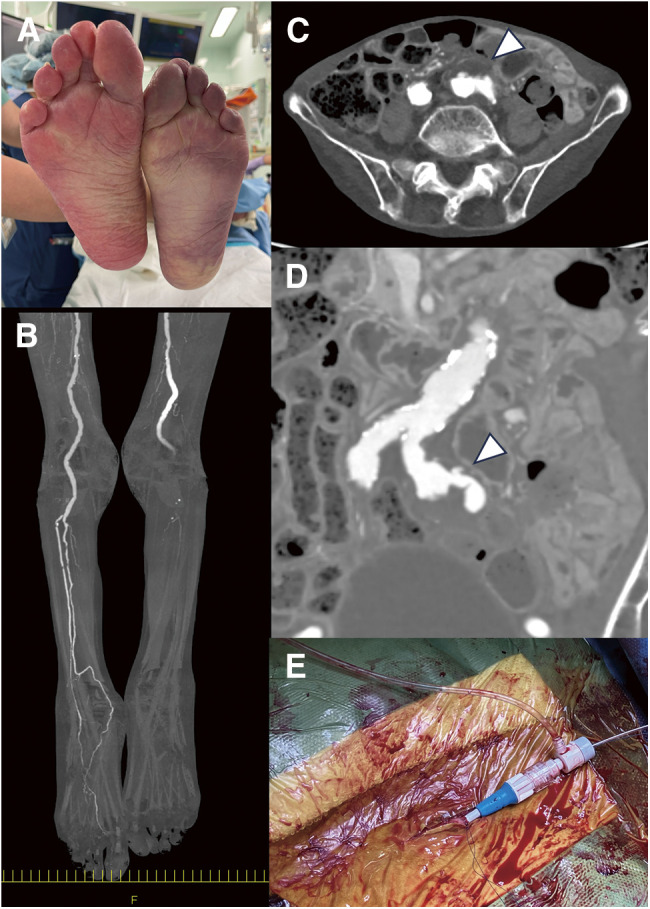
Fig. 1 Cyanotic alteration in the left foot (**A**). CTA revealed an acute occlusion in the left lower extremity artery, distal to the popliteal artery, and underlying arterial lesions in the right infrapopliteal arteries (**B**). The left CIAA with a thrombus was identified as the source of the embolism (**C** and **D**). The left CFA was exposed and an 8-French sheath was inserted (**E**). CTA: computed tomography angiography; CFA: common femoral artery; CIAA: common iliac artery aneurysm

A 96-year-old male, residing in a nursing home, complained about pain in his left foot in the morning, prior to visiting our outpatient clinic. The left foot exhibited cyanosis (**Fig. 1A**). Although arterial pulsation was confirmed in the left groin and popliteal fossa, the arteries in the left foot were not palpable. Furthermore, no Doppler sound was detected. He experienced paresthesia in his left foot. His left knee had been slightly restricted in movement since he had been living with a wheelchair for a long time. The ankle-brachial index (ABI) was 0.89 on the right and 0.33 on the left lower extremity, with a flat wave. The blood test revealed an elevated creatine kinase level of 1287 U/L, and the blood urea nitrogen/serum creatine ratio was 30.6/1.66 mg/dL. The electrocardiogram displayed a sinus rhythm. Enhanced computed tomography angiography (CTA) revealed a left arterial occlusion distal to the popliteal artery and underlying arterial lesions in both lower extremity arteries (**Fig. 1B**). A left common iliac artery aneurysm (CIAA) with a diameter of 33 mm and a thrombus were also detected by the CT scan (**Figs. 1C**–**1D**). He was diagnosed with left ALLI, classified as Rutherford category II-a, due to embolization from the left CIAA. Although primary amputation may be suggested due to his daily activity level, revascularization was considered for the following reasons: 1) the time from onset was 6 hours; 2) treatment of CIAA, a source of embolization, was necessary to prevent further embolic events; and 3) he could tolerate treatment under regional anesthesia. Emergency revascularization for ALLI and endovascular treatment for left CIAA were recommended. In this case, catheter-based aspiration was favored over surgical thrombectomy because it minimally exposed the artery. Moreover, aspiration was a more secure method for the thrombus of infrapopliteal arteries, accommodating body movement and restricting movement of his knee joint. We chose to utilize IS-CDTA. The ischemic limb underwent revascularization before the CIAA treatment.

**Figure 2:**
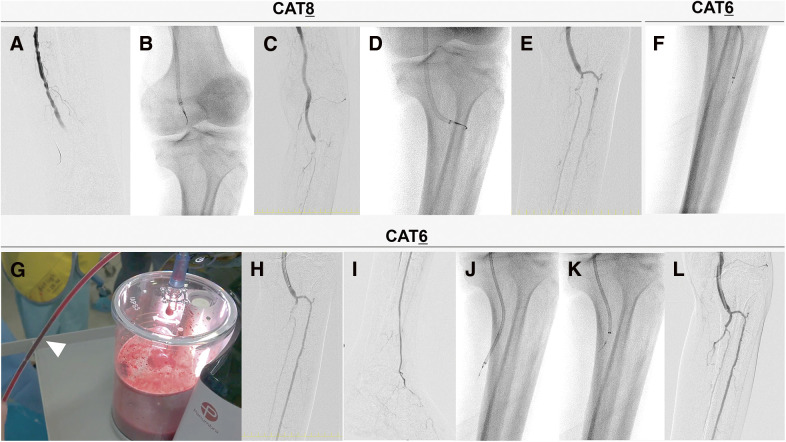
Fig. 2 Direct Thrombus Aspiration using the Indigo System Catheter. Perform angiography prior to aspiration (**A**). The thrombus was aspirated using CAT8 and Separator 8 (**B** and **C**), and the femoropopliteal segment was revascularized (**C**). The CAT8 did not cross into the ATA (**D**). Residual thrombus was present in the infrapopliteal arteries (**E**). The CAT6 was smoothly inserted into the ATA, and the thrombus was aspirated with Separator 6 (**F**). It was then identified in a tube connected to the engine (arrowhead, **G**). The ATA and DPA were successfully revascularized (**H** and **I**). The posterior tibial artery and the peroneal trunk were also treated (**J**, **K**, and **L**). ATA: anterior tibial artery; DPA: dorsalis pedis artery

**Figure 3:**
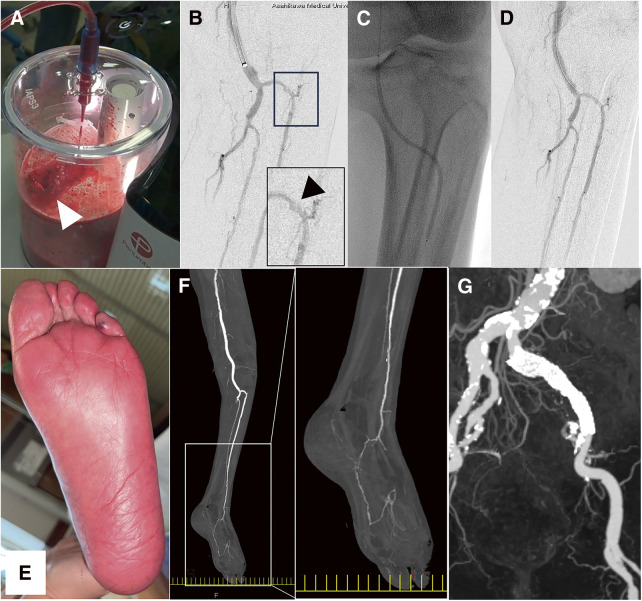
Fig. 3 The thrombus was effectively aspirated and captured in the engine (arrowhead, **A**). Posttreatment angiography of the CIAA revealed focal stenosis at the proximal ATA (indicated by the arrowhead, **B**). The arterial lesion was addressed with balloon angioplasty (**C** and **D**). His left leg was successfully salvaged (**E**), and postoperative CTA revealed well-revascularized arteries in the lower extremity (**F**) and left CIAA was covered with the stentgraft to prevent further embolization (**G**). CIAA: common iliac artery aneurysm; ATA: anterior tibial artery

The operation was conducted under regional anesthesia and sedation. The left common femoral artery (CFA) was exposed, and an 8-French sheath (Medikit, Tokyo, Japan) was inserted antegradely (**Fig. 1E**). Alongside this, the CAT8, an 8-french Indigo Aspiration System catheter (Penumbra Ltd.), was advanced using a 0.035 Radifocus guidewire (Terumo, Tokyo, Japan). We identified the location of the thrombus in the distal superficial femoral artery (SFA) by aspirating blood flow from CAT8 (**Fig. 2A**). Aspiration was performed in and out of Separator 8, a component of CAT 8, ranging from the distal SFA to the distal popliteal artery (**Fig. 2B**). Intraoperative angiography revealed the occlusion of infrapopliteal arteries (**Fig. 2C**). Despite attempts to progress CAT8 into the anterior tibial artery (ATA) alongside a 0.035 Radifocus guidewire, it was too large to cross into the middle segment of the ATA (**Fig. 2D**). Thrombosis was detected in the infrapopliteal arteries (**Fig. 2E**). We downsized from CAT8 to CAT6, resulting in CAT6 crossing into the distal ATA (**Fig. 2F**). During the thrombosis aspiration for ATA, no aspiration flow was observed to the Penumbra engine due to the CAT6 being completely wedged in the proximal ATA. However, an aspirated thrombus was identified in a tube connected to the Penumbra engine (**Fig. 2G**). Five micrograms of prostaglandin E1 were directly injected into the ATA following aspiration. Arterial flow to the ATA and dorsalis pedis artery (DPA) was restored (**Figs. 2H**–**2I**). The thrombus of the peroneal trunk and that of the posterior tibial artery were also aspirated. This was possible because the 0.035 Radifocus guidewire was able to cross them, and the CAT6 was smoothly inserted into them (**Figs. 2J**-**2K**). Prostaglandin E1 was also administered to them. Angiography revealed that the arterial flow of the ATA and peroneal trunk was revascularized (**Fig. 2L**). The thrombus was effectively aspirated and captured in the engine (**Fig. 3A**). The 8-French sheath was removed, and a 12-French sheath was retrogradely inserted into the CFA following the insertion of the 8-French sheath. Gore Excluder contralateral leg (PLC121000J; W. L. Gore & Associates, Newark, DE, USA) was positioned in the common iliac artery and external iliac artery, without embolization of the left internal iliac artery. This approach was taken to save time and prevent further embolization. Following the endovascular therapy for CIAA, angiography revealed slow arterial flow due to spastic changes in the DPA. However, no distal embolization was detected. The proximal ATA was treated with balloon angioplasty using a 2.5 × 100 mm SHIDEN (Kaneka Medics, Tokyo, Japan) due to a stenotic lesion (**Figs. 3B**–**3D**). Additionally, 5 μg of prostaglandin E1 was injected to enhance arterial flow. Blood loss was 695 mL during surgery, including 600 mL of aspiration; therefore, 840 mL of blood transfusion was required intraoperatively. However, no blood transfusion was required after surgery. Postoperatively, the Doppler sound of the DPA was well confirmed. The DPA was palpable on the first postoperative day, and his leg was successfully salvaged (**Fig. 3E**). The ABI increased from 0.33 to 0.98. He was discharged on postoperative day 7 and there were no events 4 months after treatment. The postoperative CTA showed that the lower extremity arteries were well revascularized and CIAA was covered with the stent graft (**Figs. 3F**–**3G**).

## Discussion

ALLI is technically defined as ischemia affecting the lower extremities, lasting 14 days or less. The associated morbidity and mortality rates are significantly high, with reported 1-year mortality rates of 20%–40% and a 1-year limb salvage rate of 80%–90%.^2)^ The clinical outcomes of ALLI have not changed over decades due to the extreme diversity of the population. ALLI is a medical emergency that must be quickly recognized. The time constraint is due to the duration that skeletal muscle can tolerate ischemia—approximately 4 to 6 hours. The treatment approach of ALLI is heterogeneous in order to restore blood flow. Over the past few decades, endovascular techniques have rapidly evolved, gradually replacing traditional open surgical strategies like embolectomy and bypass surgery. The range of revascularization strategies can vary from catheter-directed thrombolysis (CDT) to surgical thromboembolectomy. Catheter-based therapy has a superior survival rate compared to surgical interventions.^3)^ CDT is a major treatment for ALLI. It achieved approximately an 80% high direct technical success rate, and 89% of patients survived without amputation within 30 days. However, the high rate of CDT-related complications, such as major bleeding, is a significant disadvantage of CDT.^4)^ In this regard, mechanical aspiration offers various advantages, including safety and efficacy, for ALLI treatment. IS-CDTA is a viable choice for a quick and percutaneous treatment of ALLI, significantly reducing the time from symptom onset to catheter-based treatment.^5)^ The INDIAN UP trial, which included 250 patients with ALLI treated by IS-CDTA, demonstrated a high rate of technical success and good arterial flow (91%). No systemic bleeding complications or serious adverse events related to the device were reported. The 30-day survival rate was 97%, and the limb salvage rate was 98%.^6)^

IS-CDTA facilitates active thrombectomy by utilizing a vacuum pump that produces significant suction, allowing for the aspiration of clots of various sizes and lengths. IS-CDTA has two primary benefits: it eliminates the need for lytics, and it ensures immediate flow restoration. The use of this treatment when thrombolysis is contraindicated or has failed is already well-established. In the future, it may likely become the first-line endovascular option for patients with ALI. IS-CDTA consists of three components: an aspiration catheter, a separator, and an engine that functions as a pump. ALLI offers four different sizes of aspiration catheters: CAT3, CAT5, CAT6, and CAT8 (ranging from 3.4- to 8.0-French gauge in distal outer diameter). CAT8 is categorized into three different types based on tip shape or catheter length. They can be appropriately utilized with the treated vessel’s size and direction. The separators are designed to mobilize the clot and clean the catheter lumen, thereby restoring flow for continuous aspiration. The separator has four different sizes for ALLI. SEP3, SEP5, SEP6, and SEP8 are each available in the size of CAT. The system employs an engine that can provide an almost pure, continuous vacuum (−29 inHg or 98.2 kPa) to the catheters, facilitating the removal of thrombus in vessels of different sizes. Reasons of indication of IS-CDTA alternative to conventional thromboembolectomy for the present case were as follows: 1) Regional anesthesia was given to the patient and minimum dissection of puncture site without exposure of SFA and deep femoral artery, 2) left knee joint contracture might disturb conventional thrombectomy even if a guidewire was used, and 3) IS-CDTA was more appropriate alternative compared to the conventional thromboembolectomy for involuntary movement as well as the two reasons because of that IS-CDTA was a catheter-based treatment using CAT8 or CAT6, which were secured sheaths, beyond the knee joint to treat infrapopliteal artery occlusion. A larger size provides a more powerful vacuum force, indicating that CAT8 can be used for the occlusion of femoropopliteal segments. Therefore, an 8-French sheath is typically inserted at the puncture site. In the present case, the CFA was surgically exposed with minimum manipulation for the treatment of ALLI and CIAA. The CAT8 was effective in aspirating thrombus from the femoropopliteal arterial segment. However, it was too large to cross the infrapopliteal arteries. In contrast, the CAT6 was successful in aspirating thrombus located in the infrapopliteal arteries.

Factors associated with 1-year amputation-free survival (AFS) include severe grade of ALI, technical failure, and occlusion of the infrapopliteal artery.^7)^ Technical failures occur at a rate of 20%–30%, regardless of the method of revascularization. Especially, infrapopliteal artery occlusion is associated with AFS and technical failure, indicating that anatomically, infrapopliteal arterial occlusion is difficult to manage even with an endovascular approach. Catheter-based treatment is effective for occlusion; however, 36% of patients required additional balloon angioplasty for underlying arterial lesions. This resulted in a major amputation rate of 14%.^8)^ Endovascular and surgical approaches yield similar and acceptable limb salvage and survival rates. However, the involvement of tibial arteries was significantly negatively associated with surgical thromboembolectomy.^9^^,^^10)^ In this context, IS-CDTA could be effectively applied to infrapopliteal lesions, which are a negative factor for AFS in patients with ALLI.^7)^ In a study involving ALLI due to aortoiliac and/or femoropopliteal artery occlusion, endovascular treatment may be the preferred method for patients with Rutherford class I and IIa of ALI.^11)^ The European Society for Vascular Surgery also recommended considering percutaneous CDT as an alternative to surgery for patients with Rutherford grade IIa ALLI.^12)^ The INDIAN trial, which studied the safety and efficacy of IS-CDTA in patients with ALLI, demonstrated acceptable outcomes. This was despite the involvement of occlusion in infrapopliteal arteries associated with ALLI, accounting for 48% of below-the-knee thrombus in all ALLI cases. Adjunctive therapy was effective in increasing the technical success rate for underlying arterial lesions (26%) and residual thrombosis (35%) following IS-CDTA, as demonstrated in the current case.^1)^ The four societies related to Japanese medicine have introduced IS-CDTA as an alternative to urokinase for treating ALLI, DVT, and SMA occlusions. They established rules for using IS-CDTA if surgical thromboembolectomy failed. Occlusion of femoropopliteal artery segment is treated by surgical thromboemboloectomy; however, sometimes we had difficulty of thromboembolectomy for infrapopliteal artery occlusion with arteriosclerotic lesions since thromboembolectomy did not always remove all clots because of underlying arterial lesions. Conduction of thromboembolectomy and angioplasty for thrombus occlusion of diseased segment of infrapopliteal arteries was crucial when done simultaneously. In these cases, urokinase was effective to dissolve residual blood clots after thromboembolectomy. IS-CDTA could be an alternative to treat occlusion of underlying arterial disease after supply disturbance of urokinase. As illustrated above, surgical thromboembolectomy is not preferred for infrapopliteal artery occlusion with an underlying arterial lesion, however it is still the first option for ALLI. IS-CDTA is a new option for cases with anatomical complexity and cases unsuitable for surgery to treat acute ischemic limbs in Japan.

## Conclusion

A 96-year-old male, diagnosed with left ALLI due to embolization from left CIAA, was successfully treated with IS-CDTA. This was the first such case in Japan. IS-CDTA possesses a low-invasive property, making it an alternative to urokinase thrombolysis for ALLI treatment.

## Informed Consent

The patient provided written consent at the time of discharge for his information to be used for research and publication.

## Acknowledgments

The authors wish to thank Hideaki Obara from JSVS, Makoto Mo from JSP, Hiroyoshi Yokoi from CVIT, and Hiroyuki Tajima from JSIR for every effort of introduction of the device. This study was supported by Asahikawa Medical University Surgical Educational Support Organization (AMUSE).

## Disclosure Statement

All authors have no conflict of interest.

## Author Contributions

Treatment conception: SK and NA

Surgical procedures: SK, SO, and NA

Patient management: SK, TD, KK, and NK

Manuscript preparation: SK and NA

Critical review and revision: all authors

Final approval of the article: all authors

Accountability for all aspects of the work: all authors.
